# The Predictive Value of Dynamic Intrinsic Local Metrics in Transient Ischemic Attack

**DOI:** 10.3389/fnagi.2021.808094

**Published:** 2022-02-10

**Authors:** Huibin Ma, Guofeng Huang, Mengting Li, Yu Han, Jiawei Sun, Linlin Zhan, Qianqian Wang, Xize Jia, Xiujie Han, Huayun Li, Yulin Song, Yating Lv

**Affiliations:** ^1^School of Information and Electronics Technology, Jiamusi University, Jiamusi, China; ^2^Integrated Medical School, Jiamusi University, Jiamusi, China; ^3^Key Laboratory of Intelligent Education Technology and Application of Zhejiang Province, Zhejiang Normal University, Jinhua, China; ^4^Department of Neurology, The First Affiliated Hospital, Dalian Medical University, Dalian, China; ^5^Department of Neurology, Anshan Changda Hospital, Anshan, China; ^6^Faculty of Western Languages, Heilongjiang University, Harbin, China; ^7^School of Teacher Education, Zhejiang Normal University, Jinhua, China; ^8^Center for Cognition and Brain Disorders, The Affiliated Hospital of Hangzhou Normal University, Hangzhou, China

**Keywords:** resting-state fMRI, dynamic local metric, machine learning, support-vector machine, transient ischemic attack

## Abstract

**Background:**

Transient ischemic attack (TIA) is known as “small stroke.” However, the diagnosis of TIA is currently difficult due to the transient symptoms. Therefore, objective and reliable biomarkers are urgently needed in clinical practice.

**Objective:**

The purpose of this study was to investigate whether dynamic alterations in resting-state local metrics could differentiate patients with TIA from healthy controls (HCs) using the support-vector machine (SVM) classification method.

**Methods:**

By analyzing resting-state functional MRI (rs-fMRI) data from 48 patients with and 41 demographically matched HCs, we compared the group differences in three dynamic local metrics: dynamic amplitude of low-frequency fluctuation (d-ALFF), dynamic fractional amplitude of low-frequency fluctuation (d-fALFF), and dynamic regional homogeneity (d-ReHo). Furthermore, we selected the observed alterations in three dynamic local metrics as classification features to distinguish patients with TIA from HCs through SVM classifier.

**Results:**

We found that TIA was associated with disruptions in dynamic local intrinsic brain activities. Compared with HCs, the patients with TIA exhibited increased d-fALFF, d-fALFF, and d-ReHo in vermis, right calcarine, right middle temporal gyrus, opercular part of right inferior frontal gyrus, left calcarine, left occipital, and left temporal and cerebellum. These alternations in the dynamic local metrics exhibited an accuracy of 80.90%, sensitivity of 77.08%, specificity of 85.37%, precision of 86.05%, and area under curve of 0.8501 for distinguishing the patients from HCs.

**Conclusion:**

Our findings may provide important evidence for understanding the neuropathology underlying TIA and strong support for the hypothesis that these local metrics have potential value in clinical diagnosis.

## Introduction

Transient ischemic attack (TIA) is a transient neurological dysfunction triggered by focal brain, medulla spinalis, or retinal ischemia, also known as “small stroke” (Easton et al., 2009). As the problem of population aging becomes more and more serious, the death rate due to stroke is constantly increasing. Preventing the social harm caused by stroke is very important (On et al., 2021). Evidence presented in previous studies has demonstrated a fact that TIA can be regarded as one of the main, under-recognized, and modifiable risk factors for stroke (Rothwell and Warlow, 2005; Turner et al., 2019). Given up to 80% of strokes after TIA are preventable (Coutts, 2017), accurate diagnosis of TIA is valuable and meaningful from the perspective of offering the greatest opportunity for the early intervention of stroke. However, the transient symptoms make the diagnosis of TIA hard and difficult. Therefore, objective and reliable biomarkers are urgently needed in clinical practice.

Resting-state functional magnetic resonance imaging (rs-fMRI), which measures the changes in the blood oxygen level-dependent (BOLD) signals, is a promising tool to explore the functional alterations of human brain (Biswal et al., 1995; Fox and Raichle, 2007). Several methods have been proposed and proven to be effective in characterizing the local features of the brain function, such as the amplitude of low-frequency fluctuations (ALFF), which measures signal strength in low-frequency oscillations (LFOs) of local spontaneous neural activity (Zang et al., 2007); fractional ALFF (fALFF), which characterizes the relative contribution of a specific LFO to the entire frequency range (Zou et al., 2008); and regional homogeneity (ReHo), which reflects the coherence of local neural activity among spatially neighboring regions (Zang et al., 2004). These three methods can reveal local brain activity from different perspectives and have been widely applied to localize the functional abnormalities in brain disorders (Zang et al., 2007; Gupta et al., 2020; Li et al., 2021). With regard to TIA, it has been shown that TIA is associated with the reductions of ReHo in the right dorsolateral prefrontal cortex, inferior prefrontal cortex, ventral anterior cingulate cortex, and dorsal posterior cingulate cortex (Guo et al., 2014) and decreased ALFF in the left middle temporal gyrus (Lv et al., 2019). These studies indicated that local metrics are promising to locate abnormal brain areas for TIA; however, only one value for each metric was calculated for the entire rs-fMRI scan, which ignored the characteristics of the dynamic brain variation or time-varying process of the BOLD signal along the course during fMRI scanning (Liao et al., 2015; Deng et al., 2016). In fact, previous studies have suggested that brain activity exhibits dynamic characteristics over time-varying process (Sporns, 2011; Abrams et al., 2013; Yin et al., 2013; Bassett and Sporns, 2017). Brain dynamics are thought to reflect the functional capacity of the neural system and could offer physiological neuromarker in many neurological and psychiatric diseases (Damaraju et al., 2014; Liao et al., 2014). Thus, it is of great importance to explore the dynamic changes in these local metrics. Moreover, the traditional identification of TIA, which was mainly judged from subjective evaluation of the symptoms, is time-consuming and labor-intensive with relatively low accuracy rate. Therefore, whether these local abnormalities could serve as objective and reliable biomarkers for TIA is still need to be clarified.

The support-vector machine (SVM) is a supervised machine learning algorithm that aims to maximize the margin so as to classify data points between classes in a high-dimensional space (Pereira et al., 2009) and has been widely used to assist diagnosis of neurological disorders. For example, Bu et al. (2019) selected the optimal features from the ALFF, fALFF, ReHo, and degree centrality of different brain regions and applied SVM to differentiate patients with obsessive-compulsive disorder from healthy controls (HCs). In a previous study, excellent performance with an accuracy of 95.37% was achieved when ALFF maps were employed, followed by ReHo, fALFF, and DC. Ma X. et al. (2020) chose 54 amyotrophic lateral sclerosis participants and used ALFF and d-ALFF as the SVM classification feature, and the classification accuracy was 79.63%. From what have been listed above, a reliable conclusion can be drawn that SVM has relatively high accuracy compared with traditional methods to differentiate the patients from healthy subjects. Hence, we used SVM to examine whether local abnormalities can be used as diagnostic and prognostic indicators for TIA.

In this study, we first employed sliding window approaches (Chen et al., 2018; Duncan and Small, 2018) to investigate the dynamic changes of three resting-state local metrics: dynamic ALFF (d-ALFF), dynamic fALFF (d-fALFF), and dynamic ReHo (d-ReHo) in patients with TIA. By using the SVM classification method, we further examined whether these dynamic local abnormalities could differentiate patients from HCs. We used two hypotheses in this study: (i) patients with TIA would exhibit significant temporal variability compared with HCs and (ii) the d-ALFF, d-fALFF, and d-ReHo values could be sensitive biomarkers to distinguish patients with TIA from HCs.

## Materials and Methods

### Participants

Data were obtained from 51 suspected patients with TIA from the Department of Neurology, Anshan Changda Hospital from April 2015 to June 2016. The patients with transient neurological symptoms had been evaluated to have a possible vascular etiology judged by recruited clinical neurologists. Patients who have history of hemorrhage, leukoaraiosis, migraine, epilepsy, or psychiatric diseases were excluded in this study. Information about each participant was recorded as follows: (Easton et al., 2009) history of TIA and stroke; (On et al., 2021) previous risk factors, such as hypertension, diabetes mellitus, coronary artery disease, current smoking, and drinking behavior; (Turner et al., 2019) medications used ahead of the MRI scanning; (Qiu and Xu, 2020) in hospital evaluation of arterial stenosis (carotid duplex ultrasound and MR angiography), atrial fibrillation (ECG), and brain infarcts (diffusion-weighted imaging and T2-FLAIR); and (Rothwell and Warlow, 2005) 1-year telephone follow-up of stroke and/or TIA attack. In addition, four patients dropped out during the 1-year follow-up period. The risk of each patient for subsequent stroke was evaluated by age, blood pressure, clinical features, duration of symptoms, and history of diabetes (ABCD2) (Johnston et al., 2007) score.

Besides, the study involves forty-one age- and sex-matched HCs with no physical diseases or history of psychiatric or neurological disorders from local community through advertising.

Finally, three patients were excluded because of unacceptable image quality of multimodal MRI data (incomplete coverage of the whole brain in rs-fMRI scan or missing 3D T1 image), leaving 48 patients with TIA and 41 HCs in the final analysis. Of the 48 patients, 25 patients suffered TIA (not first-time attack) and 4 patients suffered stroke. Detailed demographic and clinical information of all participants are displayed in Table 1.

**TABLE 1:** Demographic and clinical information.

	TIA (*n* = 48)	HCs (*n* = 41)	*p* value
Age (year, mean ± SD)	57.6 ± 9.8	55.0 ± 8.0	0.182^t^
Gender (male/female)	37/11	30/11	0.670^χ^
FD (mean ± SD)	0.06 ± 0.03	0.06 ± 0.05	0.961^t^
MMSE (mean ± SD)	29.2 ± 2.6	28.6 ± 1.7	0.222^t^
Blood systolic pressure (mmHg, mean ± SD)	145.5 ± 20.8	126.9 ± 19.8^a^	<0.001^t^
Blood diastolic pressure (mmHg, mean ± SD)	86.7 ± 10.4	80.0 ± 10.9^a^	0.007^t^
Blood sugar level (mmol/L, mean ± SD)	6.3 ± 2.1	5.2 ± 0.7^a^	<0.001^t^
Total cholesterol (mmol/L, mean ± SD)	5.3 ± 1.2	4.8 ± 1.0^a^	0.037^t^
Triglycerides (mmol/L, mean ± SD)	1.6 ± 0.9	1.9 ± 1.3^a^	0.234^t^
HDL-C (mmol/L, mean ± SD)	1.1 ± 0.2	1.1 ± 0.3^a^	0.311^t^
LDL-C (mmol/L, mean ± SD)	3.3 ± 1.0	2.7 ± 0.9^a^	0.004^t^
ABCD2 scores (median)	4 (2–6)		

*^t^Data were obtained using two-sample two-side t-tests; ^χ^ Data were obtained using Pearson’s chi-square tests; ^a^Data were missing for 6 controls; TIA, transient ischemic attack; HCs, healthy controls; MMSE, mini-mental state examination; HDL-C, high-density lipoprotein cholesterol; LDL-C, low-density lipoprotein cholesterol; DWI, diffusion-weighted imaging; FD, frame-wise displacement.*

### Physiological and Biochemical Tests

All participants completed blood systolic pressure, blood diastolic pressure, blood sugar level, total cholesterol, triglycerides, high-density lipoprotein cholesterol (HDL-C), and low-density lipoprotein cholesterol (LDL-C) physiological/biochemical tests within 24 h before the MRI data acquisition. Additionally, all participants underwent the mini-mental state examination (MMSE) (Mingyuan, 1998) to evaluate global cognition.

### Data Acquisition

GE MR-750 3.0 T scanner (GE Medical Systems, Inc., Waukesha, WI, United States) was used. The time interval between the latest TIA attack and subsequent MRI scanning was 0.25–16 days for the patients. During the resting state scanning, all participants were instructed to refrain from any cognitive task (Biswal et al., 1995). Specifically, all participants were required to keep relaxed, to close their eyes but not fall asleep, not to think systematically, and to remain motionless.

BOLD-fMRI EPI (echo planar imaging) scan parameters included TE (echo time) = 30 ms, TR (repetition time) = 2,000 ms, FA (flip angle) = 60°, matrix size = 64 × 64, thickness/gap = 3.2/0 mm, slices = 43, time = 8 min. A total of 240 scans were collected.

The high-resolution anatomic 3D T1 sequence had the following parameters: 176 sagittal slice, TR = 8,100 ms, TE = 3.1 ms, matrix = 256 × 256, voxel size = 1 mm × 1 mm × 1 mm, thickness/gap = 1/0 mm. This session lasted for about 5 min.

### Data Preprocessing

Resting-state fMRI images and structural images were preprocessed using the Temporal Dynamic Analysis (TDA) toolbox based on RESTplus version 1.24 (Jia et al., 2019)^1^ running on Matlab2014a (MathWorks, Natick, MA, United States) and included the following steps: (Easton et al., 2009) the first 10 time points were removed to make the initial MRI signal reach steady state and to permit the participants to adapt to the scanning environment, and the remaining 230 consecutive volumes were used for data analyses; (On et al., 2021) slice timing and head motion were done in the left volumes of images, and no participant had a head movement bigger than 3 mm or rotation larger than 3°; (Turner et al., 2019) spatial normalization to the Montreal Neurological Institute space *via* the deformation fields derived from tissue segmentation of structural images was performed, and all images were then resampled into 3 mm × 3 mm × 3 mm voxels; (Qiu and Xu, 2020) for the dynamic ALFF and fALFF calculations, spatial smoothing (4 mm isotropic Gaussian kernel) was performed; (Rothwell and Warlow, 2005) detrending was used to correct the signal drift in real time; (Coutts, 2017) nuisance covariate regression (head motion effect using Friston 24 parameter model) from fMRI data (Friston et al., 1996) was calculated; and (Biswal et al., 1995) for the dynamic ReHo calculations, band-pass filtering (0.01–0.08 Hz) was applied to reduce low-frequency drift and high-frequency noise. The band-pass filter was applied only in ReHo.

### Dynamic Measurements

Dynamic local metrics analysis was performed using TDA toolkits based on RESTplus (Jia et al., 2019) (see text footnote 1). The dynamic metrics was calculated using a sliding window method, and it is sensitive in detecting time-dependent variations and examining metrics variability over the whole brain (Hindriks et al., 2016; Yan et al., 2017; Fu et al., 2018; Ma M. et al., 2020). The most important parameter in resting-state dynamic computation is window length. Previous studies have demonstrated that the minimum window length should be larger than 1/fmin (where fmin is the minimum frequency of time series) so that the spurious fluctuations could be excluded (Leonardi and Van De Ville, 2015). Therefore, we applied a sliding window length of 50 TR (100 s) and a shifting step size of 1 TR (2 s). This procedure produced a total of 180 windows for each participant. Based on these sliding windows, we proposed three local metrics, namely, d-ALFF, d-fALFF, and d-ReHo (Liao et al., 2018; Yu et al., 2019; Tian et al., 2021).

#### d-ALFF Calculation

The time courses for each individual voxel were subject to a fast Fourier transformation to the frequency domain, and the power spectrum was determined. The square root of this spectrum was calculated for each frequency and then averaged across 0.01–0.08 Hz. This averaged square root was used as an ALFF index (Zang et al., 2007). After calculating ALFF of all voxels in time windows, each participant will get several window-based ALFF maps. Then, we computed the mean and SD of each voxel in all window-based ALFF maps for each participant and further got the corresponding coefficient of variation (CV = SD/mean). The CV maps were prepared for further statistical analysis.

#### d-fALFF Calculation

The fALFF was calculated as the ratio of the amplitude within the low-frequency range (0.01–0.08 Hz) to the total amplitude over the full frequency range (0–0.25 Hz). It indicates the relative contribution of oscillations in the low-frequency range to the signal variations over the whole frequency range (Zou et al., 2008). Then, we computed the CV of each voxel in all window-based fALFF maps for each participant. The CV maps were used for further statistical analysis.

#### d-ReHo Calculation

Individual ReHo maps were generated by calculating the Kendall coefficient of concordance (KCC) of the time courses of a given voxel with those of its neighbors (26 voxels) in a voxel-wise manner (Zang et al., 2004). Then, we computed the CV of each voxel in all window-based ReHo maps for each participant. Finally, the CV maps were spatially smoothed with an isotropic Gaussian kernel of 4 mm full-width-at-half-maximum (FWHM). The spatially smoothed CV maps were used for further statistical analyses.

### Statistical Analyses

To detect the group differences in demographic variables between patients with TIA and HCs, two-sample *t*-tests and chi-square analyses were performed using Statistical Package for the Social Sciences (SPSS) software (SPSS Inc., Chicago, IL, United States). Age and clinical/physiological/biochemical characteristics between patients with TIA and HCs were compared using two-sample *t*-test. Sex difference was obtained with the Pearson’s chi-square test.

The dynamic metrics (d-ALFF, d-fALFF, and d-ReHo) of regional brain activity between patients with TIA and HCs were compared using two-sample *t*-tests on each voxel to examine the between-group differences in RESTplus software (Jia et al., 2019) (see text footnote 1). Multiple comparison correction was performed based on Gaussian random field theory (GRF, voxel-wise *p* < 0.005, cluster-wise *p* < 0.05, two-tailed).

### Feature Extraction and SVM Model Training

To evaluate whether the alterations of three dynamic metrics could serve as potential diagnostic indices for TIA, we performed machine learning analyses using SVM algorithm with the average dynamic metric values of all clusters showing significant among-group differences as the features.

Mapping non-linear data to a high dimensional feature space and finding a linear separating hyperplane to separate the two-group data are the core idea of the SVM algorithm. In this study, we used the Gaussian radial basis function kernel SVMs (RBF-SVM) (Cristianini and Shawe-Taylor, 2000), a implement in the LIBSVM software package (Pereira et al., 2009)^2^, to investigate the potential diagnostic indices of the dynamic metrics. We used gird search optimization algorithm to obtain the parameters that enable SVM to achieve optimal performance. The grid search method is the most basic parameter optimization algorithm. In essence, it divides the parameters to be searched into a grid of the same length in a certain space range according to the proposed coordinate system. Each point in the coordinate system represents a set of parameters. The C (last parameter C = 1) in the SVM was set to 2*^N^* (*N* from −4 to 4), and radial basis function kernel parameter γ (last parameter γ = 0.125) was optimized among the values of 2*^N^* (*N* from −4 to 4). These points are brought into the SVM system to verify its performance, and the point that makes the performance of the entire system the best is called the optimal parameter. In addition, a leave-one-out cross-validation (LOOCV) was applied to validate the performance of our proposed approach. It involved excluding a participant from each group for test and training the classifier using the remaining participants. This procedure was repeated for each participant to assess the overall accuracy of the SVM. To quantify the performance of classification methods, accuracy, sensitivity, and specificity were reported.

### Validation Analysis

To further test the reliability of our results for three dynamic local metrics, we reanalyzed the rs-fMRI data with three additional window lengths (25, 32, and 75 TRs) and 6 mm isotropic Gaussian kernel for spatial smoothing.

To explore the effects of head motion, we reanalyzed between-group differences of three dynamic metrics by treating mean framewise displacement (FD) (Jenkinson et al., 2002) as a covariate of no interest. To further test the effects of age and gender on our results, we also reanalyzed between-group differences with regressing age and gender out.

### Correlation Between Local Metrics and Clinical/Physiological/Biochemical Characteristics

Relationships with symptom severity were examined by extracting d-ALFF, d-fALFF, and d-ReHo values from the regions showing group differences and by correlating these values with blood systolic pressure, blood diastolic pressure, blood sugar level, total cholesterol, triglycerides HDL-C, LDL-C, MMSE, and duration time from the last TIA to MRI scanning. The correlations were considered significant at a threshold of *p* < 0.05.

## Results

### Clinical Data

Demographic and clinical information for the final 48 patients with TIA and 41 HCs is summarized in Table 1. The patients with TIA and HCs were matched in age (*p* = 0.182) and sex (*p* = 0.670). Compared with HCs, patients with TIA showed significantly higher systolic pressure (*p* < 0.001), diastolic pressure (*p* = 0.007), blood sugar level (*p* < 0.001), total cholesterol (*p* = 0.037), and LDL-C (*p* = 0.004). The median ABCD2 score for the patients with TIA was 4 (Coutts, 2017; On et al., 2021). Detailed demographics and the psychological characteristics of the two groups are shown in Table 1.

### Differences in d-ALFF, d-fALFF, and d-ReHo

**FIGURE 1:**
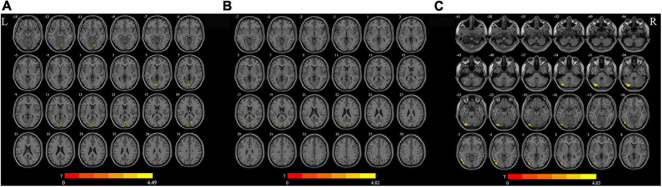
Brain regions with significant differences in d-ALFF **(A)**, d-fALFF **(B)**, and d-ReHo **(C)** between the TIA group and the HC group (after GRF correction; voxel-wise *p* < 0.005, cluster-wise *p* < 0.05, two-tailed). The color bar indicates the *T*-value. L, left; R, right; d-ALFF, dynamic amplitude of low-frequency fluctuations; d-fALFF, dynamic fractional amplitude of low-frequency fluctuations; d-ReHo, dynamic regional homogeneity.

As shown in Figure 1A, for d-ALFF, TIA increased in vermis, right calcarine, and right middle temporal gyrus. The significant differences in d-ALFF between the two groups are shown in Table 2 and Figure 1A. Compared with HCs, the patients with TIA exhibited increased d-fALFF in the opercular part of right inferior frontal gyrus and left calcarine as shown in Table 2 and Figure 1B. The cerebellum, left inferior occipital gyrus, and left inferior temporal gyrus showed increased d-ReHo in patients with TIA compared with HCs described in Table 2 and Figure 1C.

**TABLE 2:** Regions showing abnormal d-ALFF, d-fALFF, and d-ReHo in patients with TIA compared with HCs.

Metrics	Voxels	Peak MNI Coordinate (mm)			Peak T value	Effect size	Brian Regions (AAL)
		*x*	*y*	*z*			
d-ALFF	12	3	−78	−15	3.4067	0.7328	Cerebelum Vermis_6
	56	6	−75	15	4.4887	0.9655	Calcarine_R
	11	48	−66	21	4.2192	0.9075	Temporal_Middle_R
d-fALFF	12	60	12	12	4.8197	1.0367	Frontal_Inferior_Opercular_R
	47	−3	−75	9	4.3218	0.9296	Calcarine_L
d-ReHo	265	−33	−81	−30	4.0296	0.8661	Cerebellum-crus1_L
							Occipital_Inf_L
							Temporal_Inf_L

*d-ALFF, dynamic amplitude of low-frequency fluctuations; d-fALFF, dynamic fractional amplitude of low-frequency fluctuations; d-ReHo, dynamic regional homogeneity; MNI, Montreal Neurological Institute; AAL, Anatomical Automatic Labeling; L, left; R, right.*

### Classification Accuracy

**FIGURE 2:**
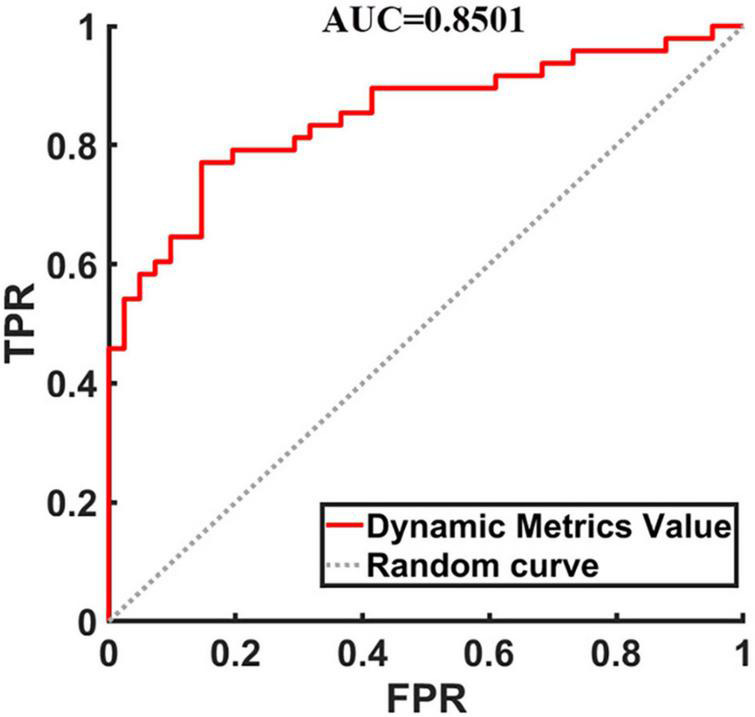
The receiver operating characteristic curve of dynamic metrics. The image of ROC was displayed using MATLAB software. FPR, false positivity rate; TPR, true positivity rate; AUC, area under the receiver operating characteristic curve.

To evaluate the classification ability of the SVM model, the accuracy, sensitivity, specificity and precision were calculated, and the receiver operating characteristic (ROC) curve of the classifier is shown in Figure 2. The curve was drawn using Receiver Operating Characteristic Assistant software (Wang et al., 2021). The performance of the classifier achieved an accuracy of 80.90%, sensitivity of 77.08%, specificity of 85.37%, precision of 86.05%, and area under curve (AUC) of 0.8501 for TIA vs. HCs.

### Validation Results

The validation analyses indicated that the TIA-related dynamic alterations in the three local metrics were consistent with the main results when using different window lengths and smooth kernels. From this perspective, smooth kernels and window lengths in our study have changed, respectively, under the condition of keeping the other calculation parameters the same, so as to fairly compare all the results and to enrich our data analysis. When the smooth kernel is 4 mm, the results of different window lengths are shown in Supplementary Tables 1–3 and Supplementary Figures 1–3, respectively. When the smooth kernel is 6 mm, the results of different window lengths are shown in Supplementary Tables 4–6 and Supplementary Figures 4–6, respectively. They are provided in the Supplementary Material.

Transient ischemic attack-related alterations in the dynamic local metrics were consistent with the main results after correcting for head motion (FD), age, and gender (refer to Supplementary Figures 7–12).

To reveal the stability of the results, SVM was also performed based on the three datasets: training set, validation set, and test set to distinguish TIA from HCs (refer to Supplementary Figure 13).

### Correlational Analysis

**FIGURE 3:**
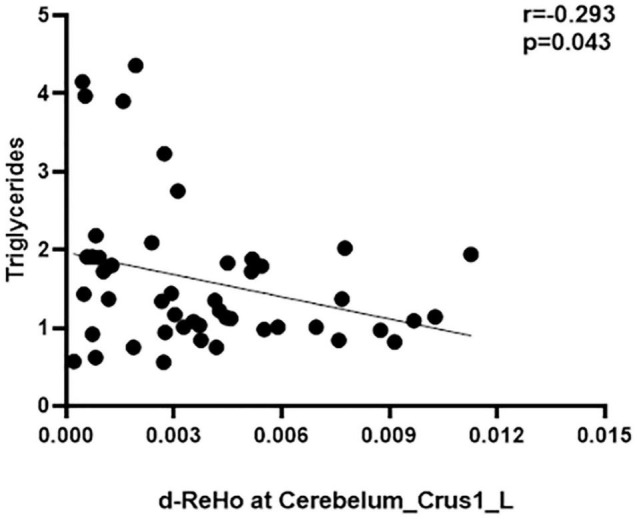
Correlation between d-ReHo variability of the Cerebelum_Crus1_L and the triglycerides score.

To avoid the influence of extreme value (value beyond 3 SD), all the correlation analyses were conducted after removing the extreme values from our data. No correlations between d-ALFF and d-fALFF different region values and clinical measures reached significance (uncorrected *p* < 0.05). The d-ReHo variability in the cerebellum was negatively correlated with the triglycerides scores of the patients with TIA (*r* = − 0.2931, *p* = 0.0432, uncorrected *p* < 0.05, Figure 3). All results of correlation between dynamic local values and clinical data are described in Supplementary Tables 7–9, and they are provided in the Supplementary Material. Here only shows the significantly related ones. There was no correlation between brain dynamic values and duration time from the last attack to scanning (refer to Supplementary Table 10 for details).

## Discussion

In this study, we used three rs-fMRI local dynamic metrics to investigate the alterations of intrinsic brain activity in patients with TIA. Compared with the HCs, the patients with TIA showed increased d-ALFF value in the cerebellum, right calcarine, and right middle temporal gyrus. Patients with TIA also exhibited increased d-fALFF value in the opercular part of the right inferior frontal gyrus and left calcarine. In addition, an increased d-ReHo value was observed in the cerebellum, left inferior occipital gyrus, and left inferior temporal gyrus. The values of d-ALFF, d-fALFF, and d-ReHo in regions that showed abnormal brain dynamics served as classification features, and the SVM classification achieved a total accuracy of 80.90%, sensitivity of 77.08%, specificity of 85.37%, precision of 86.05%, and AUC of 0.8501. Overall, these findings provide evidence for the local abnormalities in TIA, which may help to understand the neurophysiological basis and to establish objective biomarkers for TIA.

The ALFF reflects the power within the effective frequency range (0.01–0.08 Hz) and is considered as an dependable approach to detect the regional intensity of spontaneous fluctuations and to present spontaneous brain activity of the brain (Zang et al., 2007), and d-ALFF characterizes the dynamic alterations of ALFF over time (Chen et al., 2018). In this study, we found increased d-ALFF in cerebellar vermis, right calcarine, and right middle temporal gyrus in patients with TIA. Growing evidence indicates that the cerebellar vermis may contribute significantly to cognitive and global functioning in clinical populations (Steinlin, 2007; Bernard et al., 2015). Middle temporal gyrus plays an essential role in language, semantic memory processing, along with visual perception (Soderfeldt et al., 1997; Bonilha et al., 2017). The calcarine is connected with blurred visual (Yu et al., 2020). Thus, we speculated that the increased d-ALFF in these three regions was associated with the difficulties in language processing, disturbance of consciousness, and vision in patients with TIA (Lavallee and Amarenco, 2007). Notably, the abnormalities were also observed in stroke patients. For example, Chen et al. (2015) found that stroke patients exhibited larger ALFF in the right middle temporal gyrus. Jiang et al. (2019) reported decreased degree centrality in the calcarine in stroke patients. Being important risk factors for stroke, these TIA-related abnormalities already existed in the early stage of stroke according to our study.

The fALFF measures the relative spontaneous neural activity within the effective frequency range to the whole detectable frequency range, and it is calculated as the ratio of the power spectrum of the low-frequency range to that of the entire frequency range (Zou et al., 2008). Compared with ALFF, it could effectively suppress the physiological noise (Zou et al., 2008; Zuo et al., 2010), and d-fALFF examined the temporal variability of power of intrinsic brain activity and the regional features of low-frequency oscillation changes in TIA (Chen et al., 2018). In this study, the opercular part of right inferior frontal gyrus and left calcarine of patients with TIA show increased d-fALFF. A previous study has indicated that the opercular part of right inferior frontal gyrus is vital to the implementation of multicomponent behavior (Dippel and Beste, 2015). Since calcarine is related to the visual center, the damage of which also suggests the possibility of early visual center disturbance in patients with TIA (Gupta et al., 2016; Liang et al., 2020; Yu et al., 2020). The abnormality in behavior processing and disturbed vision in patients with TIA may be relevant to the increased d-fALFF in these regions (Lavallee and Amarenco, 2007). These findings indicate that the alterations of fALFF changes over time in the opercular part of right inferior frontal gyrus and left calcarine may at least partially lead to behavior impairments and visual dysfunction in patients with TIA. The speculation could be examined in future studies.

The ReHo reflects the local synchronization of spontaneous BOLD signal (Zang et al., 2004). The d-ReHo represents the change of similarity between the time series of a given voxel and its nearest neighbors (Yin et al., 2013; Avena-Koenigsberger et al., 2017). In this study, the left cerebellum-crus1, left occipital, and the left temporal showed increased d-ReHo in patients with TIA. The cerebellum is related to sensorimotor (Kansal et al., 2017) and cognitive-emotional processing (Adamaszek et al., 2017; Beckinghausen and Sillitoe, 2018). Besides, it influences motor and cognitive functions *via* cerebello-thalamocortical circuits (Middleton and Strick, 2001). The occipital lobe damage leads to visual-field loss (Tohid et al., 2015). As naming function is a critical function of temporal lobe, damage to which will result in language impairment (Trimmel et al., 2018). This result echoed the symptoms of sudden dizziness or loss of balance and coordination in patients with TIA (Lavallee and Amarenco, 2007; Easton et al., 2009; Bonilha et al., 2017). Notably, a previous study has shown decreased functional connectivity (FC) in left middle temporal gyrus within the default mode network (DMN) in patients with TIA (Li et al., 2013), decreased FC in the left middle temporal gyrus, the medial prefrontal cortex and the posterior cingulate cortex/precuneus in patients with TIA (Zhu et al., 2019), and decreased ALFF in the left middle temporal gyrus of patients with TIA (Lv et al., 2019). The decreased FC in the left middle of occipital with visual network (VN) was reported (Li et al., 2013). Taken together with these findings, it provided further evidence for the existence of impaired brain region in TIA patients, which may help to understand the pathophysiological underpinnings in patients with TIA.

Considering dynamic indicators and static indicators are bond with each other closely and these dynamic results are not reported in static results before, doing dynamic indicators within patients with TIA are urgently needed in this study, which further proved the necessity of this study. As traditional TIA diagnosis methods were subjective and lacked of clear objective standards, we used machine learning algorithms, data-driven methods used to obtain diagnostic criteria, to acquire higher reliability. Researching the algorithms of machine learning to search for the diagnosis biomarker of TIA can alleviate the contradiction between supply and demand between the limited psychiatrists with professional diagnostic qualifications and the increasing number of patients with TIA and can improve the accuracy of diagnosis and the precision of treatment at the same time. Although the application of artificial intelligence in the medical field is still in the initial stage, with more in-depth development of machine learning technology, it will become a general trend for doctors to use artificial intelligence to diagnose and manage the health of the patients in the future. SVM has been widely applied in various diseases and achieved great classification performance (Chan et al., 2019; Gui et al., 2021). Using the SVM classifier, the patients with TIA could be differentiated from HCs by dynamic local metrics. In addition, high identification accuracy of 80.90% between the TIA and HCs was achieved in this study. The results may indicate the potential value of the dynamic local metrics in the clinical diagnosis of TIA.

This is the first study to explore the dynamic characteristics of patients with TIA. In addition, the temporal brain dynamics could distinguish the patients with TIA from HCs. The results of our study are of great importance to investigate the underlying mechanism of TIA. However, considering the heterogeneity of patients, the results should be cautious when applied to the whole patients with TIA. Further studies are encouraged to pay more attention to the TIA-specific brain dynamic alterations based on this study.

This study has several potential limitations. First, although the findings were encouraging, the sample size was relatively small. The results that there were no significant correlations between TIA-related brain dynamics and clinical variables may be due to the sample size, which was demonstrated to result in low statistical power (Button et al., 2013). Accordingly, our findings should be interpreted with caution regarding the observed brain dynamic alterations as sensitive biomarkers for TIA, and further studies are required to expand the sample size to improve the statistical power. Second, although the correlation analysis revealed there was no significant correlation between TIA-related brain dynamics and clinical variables, the mismatch between two groups in clinical variables results in a heterogeneity of the sample. Third, the medications were used ahead of the MRI scanning, and it might have impact on the brain dynamics, which would be controlled in the future. Fourth, due to the lack of the TIA attack times, we could not evaluate the extent to which our findings are dependent on the attack frequency of the patients. In further studies, we would like to combine larger sample and reduce heterogeneity among participants to confirm our findings.

## Conclusion

Our results demonstrate that TIA is associated with spontaneous brain activity accompanied by dynamic characteristics, and it may provide important evidence for understanding the neuropathology underlying TIA and strong support for the hypothesis that these local metrics have a potential value in clinical diagnosis.

## Data Availability Statement

The raw data supporting the conclusions of this article will be made available by the authors, without undue reservation.

## Ethics Statement

The studies involving human participants were reviewed and approved by the Ethics Committee of the Center for Cognition and Brain Disorders, Hangzhou Normal University. The patients/participants provided their written informed consent to participate in this study.

## Author Contributions

HM, GH, ML, YS, and YL conceived and designed the experiments. GH, ML, YH, JS, QW, XH, and HL performed the experiments. HM, GH, and ML analyzed the data. HM, GH, ML, YH, LZ, and XJ participated in the completion of the manuscript. All authors have made significant scientific contributions to this manuscript and reviewed the manuscript.

## Conflict of Interest

The authors declare that the research was conducted in the absence of any commercial or financial relationships that could be construed as a potential conflict of interest.

## Publisher’s Note

All claims expressed in this article are solely those of the authors and do not necessarily represent those of their affiliated organizations, or those of the publisher, the editors and the reviewers. Any product that may be evaluated in this article, or claim that may be made by its manufacturer, is not guaranteed or endorsed by the publisher.

## Footnotes

1
http://www.restfmri.net

2
http://www.csie.ntu.edu.tw/∼cjlin/libsvm/
