# Supplementary material for: The Predictive Value of Dynamic Intrinsic Local Metrics in Transient Ischemic Attack
Source: Front Aging Neurosci. 2022 Feb 10;13:808094. doi: 10.3389/fnagi.2021.808094 (PMC8868122; doi:10.3389/fnagi.2021.808094)
Supplement: Supplementary file 1 [file Data_Sheet_1.docx]

Supplementary Materials

TABLE S1 | Regions showing abnormal d-ALFF in patients with TIA compared to HCs under different window lengths.

| Metric | Window length | Step size | Voxels | Peak MNI Coordinate (mm) | | | Peak  T value | Regions (AAL) |
| --- | --- | --- | --- | --- | --- | --- | --- | --- |
|  |  |  |  | x | y | z |  |  |
| d-ALFF | 25TR | 1TR | 29 | 0 | -84 | 6 | 4.1682 | Calcarine_L |
|  |  |  |  |  |  |  |  |  |
|  | 32TR | 2TR | 24 | 0 | -84 | 6 | 3.9342 | Calcarine_L |
|  |  |  |  |  |  |  |  |  |
|  | 50TR | 5TR | 12 | 6 | -75 | -15 | 3.4045 | Cerebelum Vermis_6 |
|  |  |  | 52 | 6 | -75 | 15 | 4.427 | Calcarine_R |
|  |  |  | 11 | 48 | -66 | 21 | 4.1783 | Temporal_Mid_R |
|  |  |  |  |  |  |  |  |  |
|  | 75TR | 20TR | 13 | -6 | -81 | -12 | 3.6729 | Lingual_L |
|  |  |  | 17 | -6 | -81 | 3 | 3.905 | Calcarine_L |

d-ALFF, dynamic amplitude of low-frequency fluctuations; TR, repetition time; MNI, Montreal Neurological Institute; AAL, Anatomical Automatic Labeling; L, left; R, right;

TABLE S2 | Regions showing abnormal d-fALFF in patients with TIA compared to HCs under different window lengths.

| Metric | Window length | Step size | Voxels | Peak MNI Coordinate (mm) | | | Peak  T value | Regions (AAL) |
| --- | --- | --- | --- | --- | --- | --- | --- | --- |
|  |  |  |  | x | y | z |  |  |
| d-fALFF | 25TR | 1TR | 10 | -21 | -96 | 0 | 4.4592 | Occipital_Mid_L |
|  |  |  | 37 | -6 | -87 | 27 | 4.505 | Cuneus_L |
|  |  |  |  |  |  |  |  |  |
|  | 32TR | 2TR | 11 | -21 | -96 | 0 | 4.334 | Occipital_Mid_L |
|  |  |  | 53 | -6 | -87 | 27 | 4.1161 | Cuneus_L |
|  |  |  |  |  |  |  |  |  |
|  | 50TR | 5TR | 42 | -3 | -75 | 9 | 4.3816 | Calcarine_L |
|  |  |  |  |  |  |  |  |  |
|  | 75TR | 20TR | 10 | 60 | 12 | 12 | 3.7991 | Frontal_Inf_Ope_R |
|  |  |  | 17 | 36 | 60 | 15 | 4.1153 | Frontal_Mid_R |

d-fALFF, dynamic fractional amplitude of low-frequency fluctuations; TR, repetition time; MNI, Montreal Neurological Institute; AAL, Anatomical Automatic Labeling; L, left; R, right;

TABLE S3 | Regions showing abnormal d-ReHo in patients with TIA compared to HCs under different window lengths.

| Metric | Window length | Step size | Voxels | Peak MNI Coordinate (mm) | | | Peak  T value | Regions (AAL) |
| --- | --- | --- | --- | --- | --- | --- | --- | --- |
|  |  |  |  | x | y | z |  |  |
| d-ReHo | 25TR | 1TR | 192 | -33 | -81 | -30 | 4.1373 | Cerebelum_Crus1_L |
|  |  |  |  |  |  |  |  |  |
|  | 32TR | 2TR | 208 | -33 | -81 | -30 | 4.1098 | Cerebelum_Crus1_L |
|  |  |  |  |  |  |  |  |  |
|  | 50TR | 5TR | 265 | -33 | -81 | -30 | 4.3816 | Cerebelum_Crus1_L |
|  |  |  |  |  |  |  |  |  |
|  | 75TR | 20TR | 250 | -45 | -78 | -3 | 3.9267 | Occipital_ Inferior_L |

d-ReHo, dynamic regional homogeneity; TR, repetition time; MNI, Montreal Neurological Institute; AAL, Anatomical Automatic Labeling; L, left; R, right;

TABLE S4 | Regions showing abnormal d-ALFF in patients with TIA compared to HCs under different window lengths.

| Metric | Window length | Step size | Voxels | Peak MNI Coordinate (mm) | | | Peak  T value | Regions (AAL) |
| --- | --- | --- | --- | --- | --- | --- | --- | --- |
|  |  |  |  | x | y | z |  |  |
| d-ALFF | 25TR | 1TR | 33 | 0 | -66 | 6 | 4.1067 | Lingual_L |
|  |  |  | 82 | 0 | -84 | 6 | 4.0083 | Calcarine_L |
|  |  |  |  |  |  |  |  |  |
|  | 32TR | 2TR | 109 | 0 | -66 | 3 | 4.0085 | Lingual_L |
|  |  |  |  |  |  |  |  |  |
|  | 50TR | 1TR | 123 | 6 | -75 | 15 | 4.4884 | Calcarine_R |
|  |  |  |  |  |  |  |  |  |
|  | 50TR | 5TR | 109 | 6 | -75 | 15 | 4.4432 | Calcarine_R |
|  |  |  |  |  |  |  |  |  |
|  | 75TR | 20TR | 34 | -6 | -72 | -3 | 3.6798 | Lingual_L |
|  |  |  | 45 | 6 | -75 | 15 | 3.9668 | Calcarine_R |

d-ALFF, dynamic amplitude of low-frequency fluctuations; TR, repetition time; MNI, Montreal Neurological Institute; AAL, Anatomical Automatic Labeling; L, left; R, right.

TABLE S5 | Regions showing abnormal d-fALFF in patients with TIA compared to HCs under different window lengths.

| Metric | Window length | Step size | Voxels | Peak MNI Coordinate (mm) | | | Peak  T value | Regions (AAL) |
| --- | --- | --- | --- | --- | --- | --- | --- | --- |
|  |  |  |  | x | y | z |  |  |
| d-fALFF | 25TR | 1TR | 20 | -36 | -87 | -6 | 4.6548 | Occipital_Inf_L |
|  |  |  | 21 | -18 | -99 | 0 | 4.237 | Occipital_Mid_L |
|  |  |  | 84 | -6 | -87 | 27 | 4.1733 | Cuneus_L |
|  |  |  |  |  |  |  |  |  |
|  | 32TR | 2TR | 30 | -18 | -99 | 3 | 3.97 | Occipital_Mid_L |
|  |  |  | 109 | 0 | -75 | 18 | 4.2633 | Cuneus_L |
|  |  |  |  |  |  |  |  |  |
|  | 50TR | 1TR | 84 | 0 | -81 | 21 | 4.3586 | Cuneus_L |
|  |  |  |  |  |  |  |  |  |
|  | 50TR | 5TR | 82 | 0 | -81 | 21 | 4.3488 | Cuneus_L |
|  |  |  |  |  |  |  |  |  |
|  | 75TR | 20TR | 20 | 36 | 60 | 15 | 4.0365 | Frontal_Mid_R |

d-fALFF, dynamic fractional amplitude of low-frequency fluctuations; TR, Repetition time; MNI, Montreal Neurological Institute; AAL, Anatomical Automatic Labeling; L, left; R, right;

TABLE S6 | Regions showing abnormal d-ReHo in patients with TIA compared to HCs under different window lengths.

| Metric | Window length | Step size | Voxels | Peak MNI Coordinate (mm) | | | Peak T value | Regions (AAL) |
| --- | --- | --- | --- | --- | --- | --- | --- | --- |
|  |  |  |  | x | y | z |  |  |
| d-ReHo | 25TR | 1TR | 364 | -33 | -81 | -30 | 4.1679 | Cerebelum_Crus1_L |
|  |  |  |  |  |  |  |  |  |
|  | 32TR | 2TR | 381 | -33 | -81 | -30 | 4.1359 | Cerebelum_Crus1_L |
|  |  |  |  |  |  |  |  |  |
|  | 50TR | 1TR | 387 | -33 | -81 | -30 | 4.0071 | Cerebelum_Crus1_L |
|  |  |  |  |  |  |  |  |  |
|  | 50TR | 5TR | 387 | -33 | -81 | -30 | 4.0436 | Cerebelum_Crus1_L |
|  |  |  |  |  |  |  |  |  |
|  | 75TR | 20TR | 368 | -51 | -75 | -12 | 3.8381 | Cerebelum_Crus1_L |

d-ReHo, dynamic regional homogeneity; TR, repetition time; MNI, Montreal Neurological Institute; AAL, Anatomical Automatic Labeling; L, left; R, right;

TABLE S7 | The relationship between d-ALFF variability in the different regions and the clinical data in the TIA patients.

| d-ALFF | Cerebelum Vermis_6 | Calcarine_R | Temporal_Middle_R |
| --- | --- | --- | --- |
| Blood sugar level | r:-0.024  p: 0.868 | r: 0.264  p: 0.073 | r: 0.094  p: 0.531 |
| Blood systolic pressure | r:-0.037  p: 0.805 | r: 0.124  p: 0.401 | r: 0.134  p: 0.365 |
| Blood diastolic pressure | r:-0.079  p: 0.594 | r:-0.056  p: 0.703 | r:-0.167  p: 0.257 |
| Total cholesterol | r:-0.170  p: 0.249 | r:-0.063  p: 0.670 | r:-0.100  p: 0.501 |
| Triglycerides | r: 0.070  p: 0.635 | r: 0.161  p: 0.28 | r: 0.228  p: 0.119 |
| HDL-C | r:-0.250  p: 0.086 | r:-0.207  p: 0.157 | r:-0.048  p: 0.745 |
| LDL-C | r:-0.107  p: 0.468 | r:-0.097  p: 0.510 | r:-0.199  p: 0.174 |
| MMSE | r:-0.240  p: 0.099 | r:-0.177  p: 0.227 | r:-0.090  p: 0.543 |

d-ALFF, dynamic amplitude of low-frequency fluctuations; HDL-C, high-density lipoprotein cholesterol; LDL-C, low-density lipoproteincholesterol; MMSE, mini-mental state examination; *p<0.05.

TABLE S8 | The relationship between d-fALFF variability in the different regions and the clinical data in the TIA patients.

| d-fALFF | Frontal_Inferior_Opercular_R | Calcarine_L |
| --- | --- | --- |
| Blood sugar level | r: 0.072  p: 0.630 | r: 0.202  p: 0.630 |
| Blood systolic pressure | r:-0.025  p: 0.863 | r: 0.062  p: 0.677 |
| Blood diastolic pressure | r:-0.247  p: 0.091 | r:-0.066  p: 0.653 |
| Total cholesterol | r: 0.091  p: 0.538 | r: 0.076  p: 0.608 |
| Triglycerides | r:-0.060  p: 0.684 | r:-0.066  p: 0.654 |
| HDL-C | r: 0.053  p: 0.719 | r:-0.002  p: 0.991 |
| LDL-C | r: 0.069  p: 0.639 | r: 0.080  p: 0.590 |
| MMSE | r:-0.155  p: 0.294 | r:-0.206  p: 0.160 |

d-fALFF, dynamic fractional amplitude of low-frequency fluctuations; HDL-C, high-density lipoprotein cholesterol; LDL-C, low-density lipoproteincholesterol; MMSE, mini-mental state examination; *p<0.05.

TABLE S9 | The relationship between d-ReHo variability in the different regions and the clinical data in the TIA patients.

| d-ReHo | Cerebellum-crus1_L |
| --- | --- |
| Blood sugar level | r:-0.030  p: 0.174 |
| Blood systolic pressure | r: 0.044  p: 0.766 |
| Blood diastolic pressure | r: 0.159  p: 0.280 |
| Total cholesterol | r:-0.206  p: 0.160 |
| Triglycerides | r:-0.293  p: 0.043* |
| HDL-C | r:-0.081  p: 0.582 |
| LDL-C | r:-0.114  p: 0.451 |
| MMSE | r:-0.042  p: 0.775 |

d-ReHo, dynamic regional homogeneity; HDL-C, high-density lipoprotein cholesterol; LDL-C, low-density lipoproteincholesterol; MMSE, mini-mental state examination; **p* < 0.05.

TABLE S10 | The spearman correlation between brain dynamic values and durations time of the last TIA attack to scanning

| **Brain dynamic values** |  | **Time of attack to scanning** |
| --- | --- | --- |
| **d-ALFF** | | |
| Cerebelum Vermis_6 | Spearman correlation (*r*) | 0.2524 |
|  | Significance (*p*) | 0.0835 |
| Calcarine_R | Spearman correlation (*r*) | -0.1455 |
|  | Significance (*p*) | 0.3238 |
| Temporal_Middle_R | Spearman correlation (*r*) | -0.0252 |
|  | Significance (*p*) | 0.865 |
| **d-fALFF** | | |
| Frontal_Inferior_Opercular_R | Spearman correlation (*r*) | -0.1653 |
|  | Significance (*p*) | 0.2615 |
| Calcarine_L | Spearman correlation (*r*) | 0.0276 |
|  | Significance (*p*) | 0.8524 |
| **d-ReHo** | | |
| Cerebellum-crus_L | Spearman correlation (*r*) | 0.0535 |
|  | Significance (*p*) | 0.7179 |

d-ALFF, dynamic amplitude of low-frequency fluctuations; d-fALFF, dynamic fractional amplitude of low-frequency fluctuations; d-ReHo, dynamic regional homogeneity


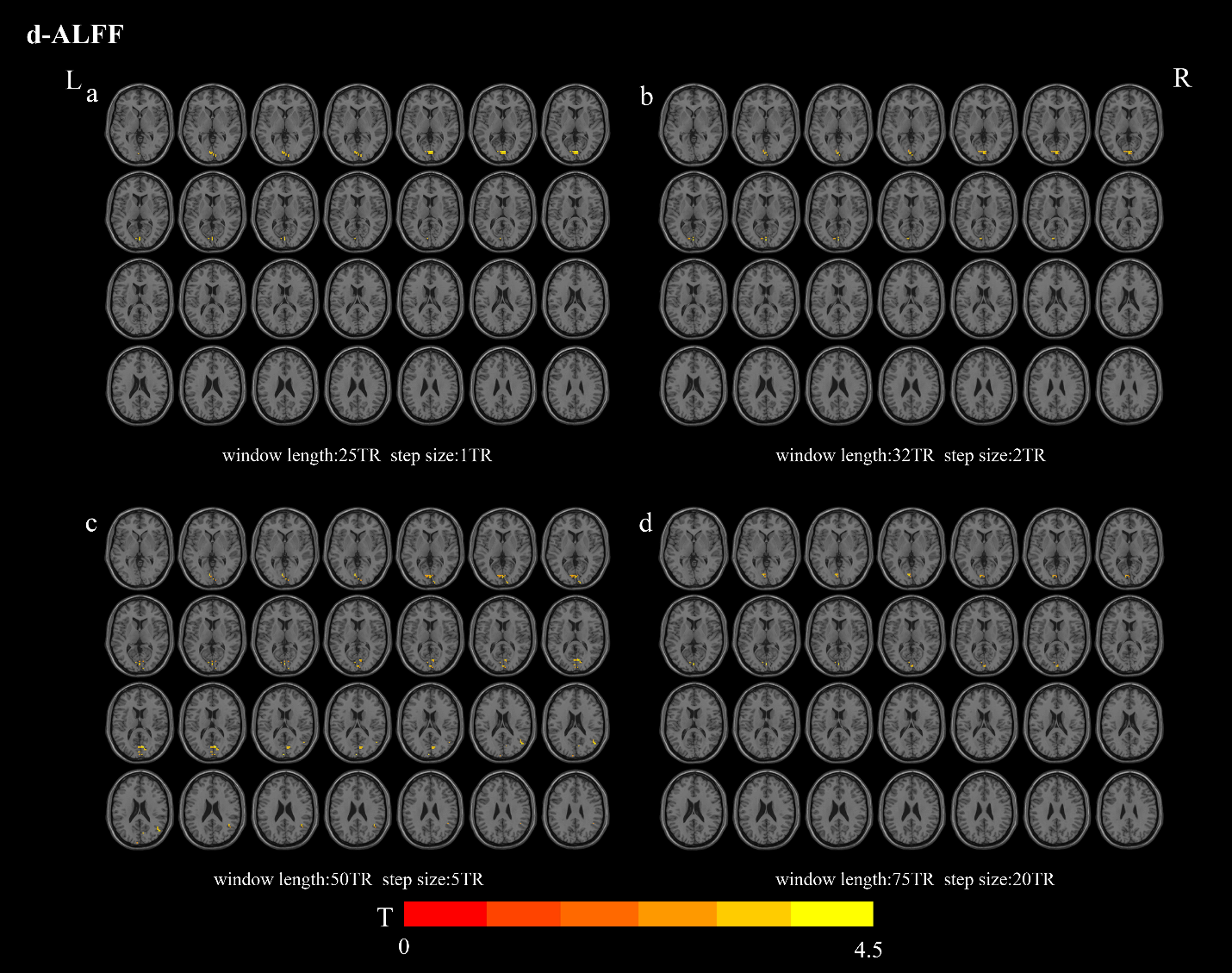


FIGURE S1 | Brain regions with significant differences in d-ALFF between TIA group and HCs group (after GRF correction; voxel-wise p < 0.005, cluster-wise p < 0.05, two-tailed) under different window lengths. Parameter: smooth kernel = 4 mm. The color bar indicates the T-value. d-ALFF, dynamic amplitude of low-frequency fluctuations; L, left; R, right.


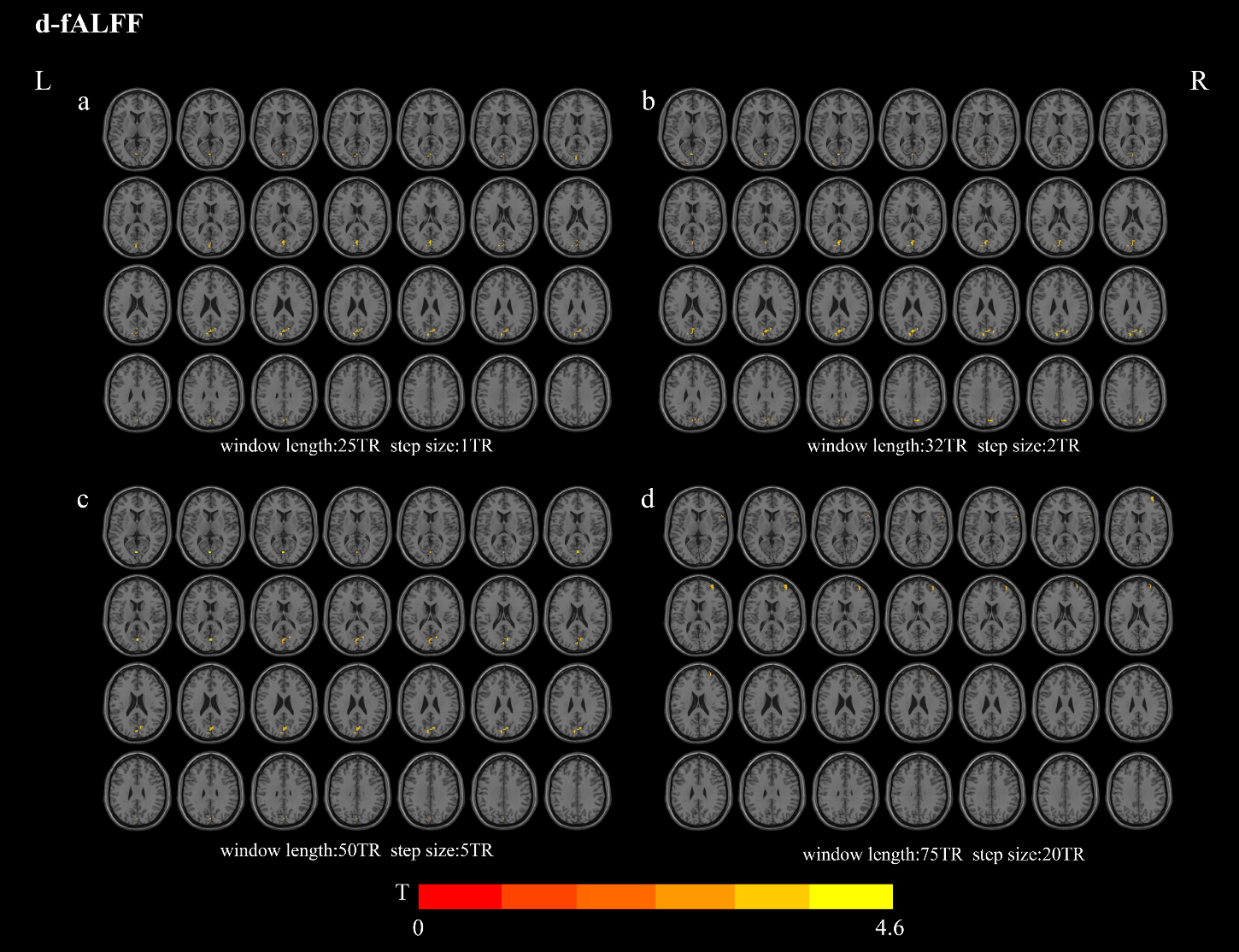


FIGURE S2 | Brain regions with significant differences in d-fALFF between TIA group and HCs group (after GRF correction; voxel-wise p < 0.005, cluster-wise p < 0.05, two-tailed) under different window lengths. Parameter: smooth kernel = 4 mm. The color bar indicates the T-value. d-fALFF, dynamic fractional amplitude of low-frequency fluctuations; L, left; R, right.


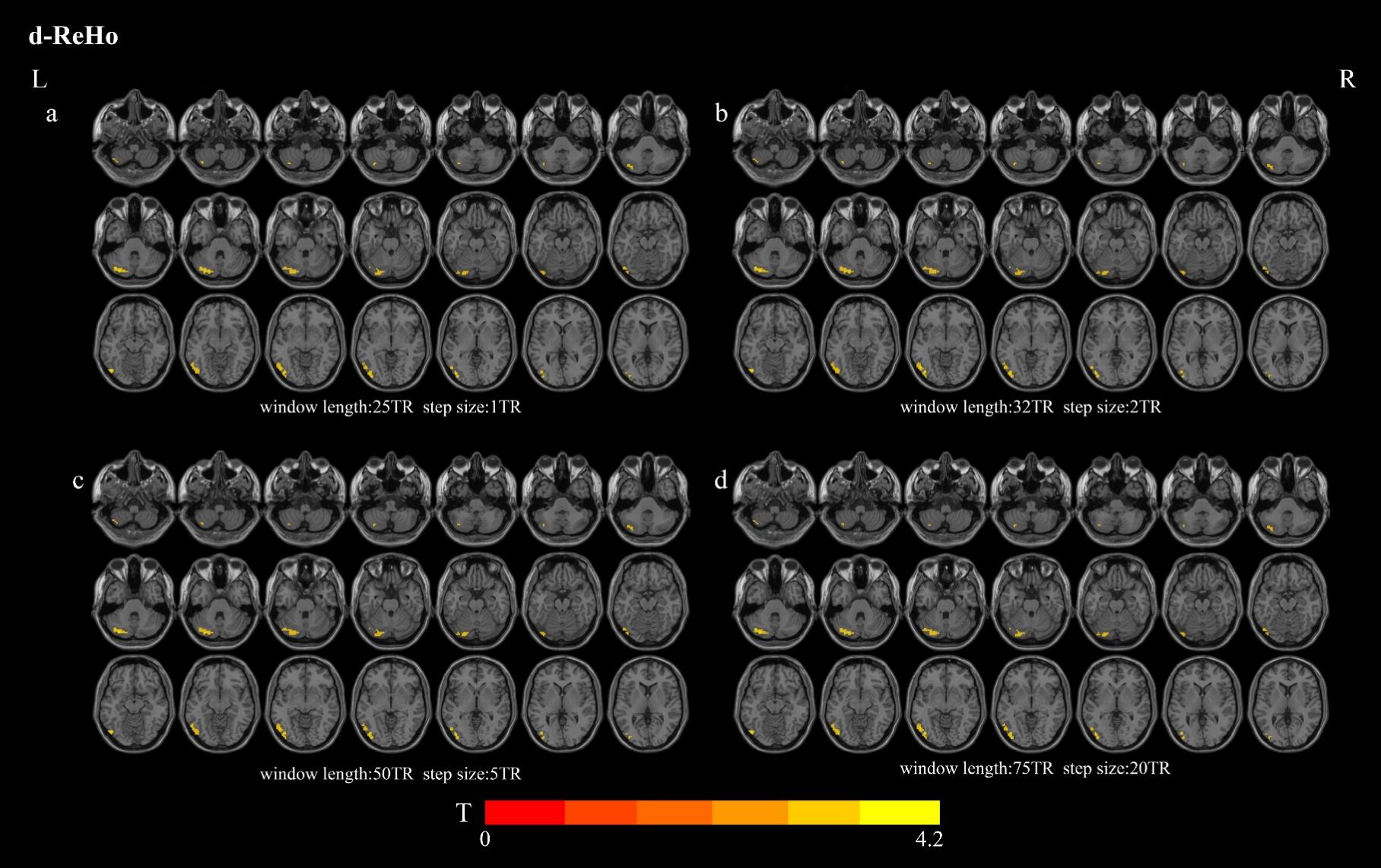


FIGURE S3 | Brain regions with significant differences in d-ReHo between TIA group and HCs group (after GRF correction; voxel-wise p < 0.005, cluster-wise p < 0.05, two-tailed) under different window lengths. Parameter: smooth kernel = 4 mm. The color bar indicates the T-value. d-ReHo, dynamic regional homogeneity; L, left; R, right.


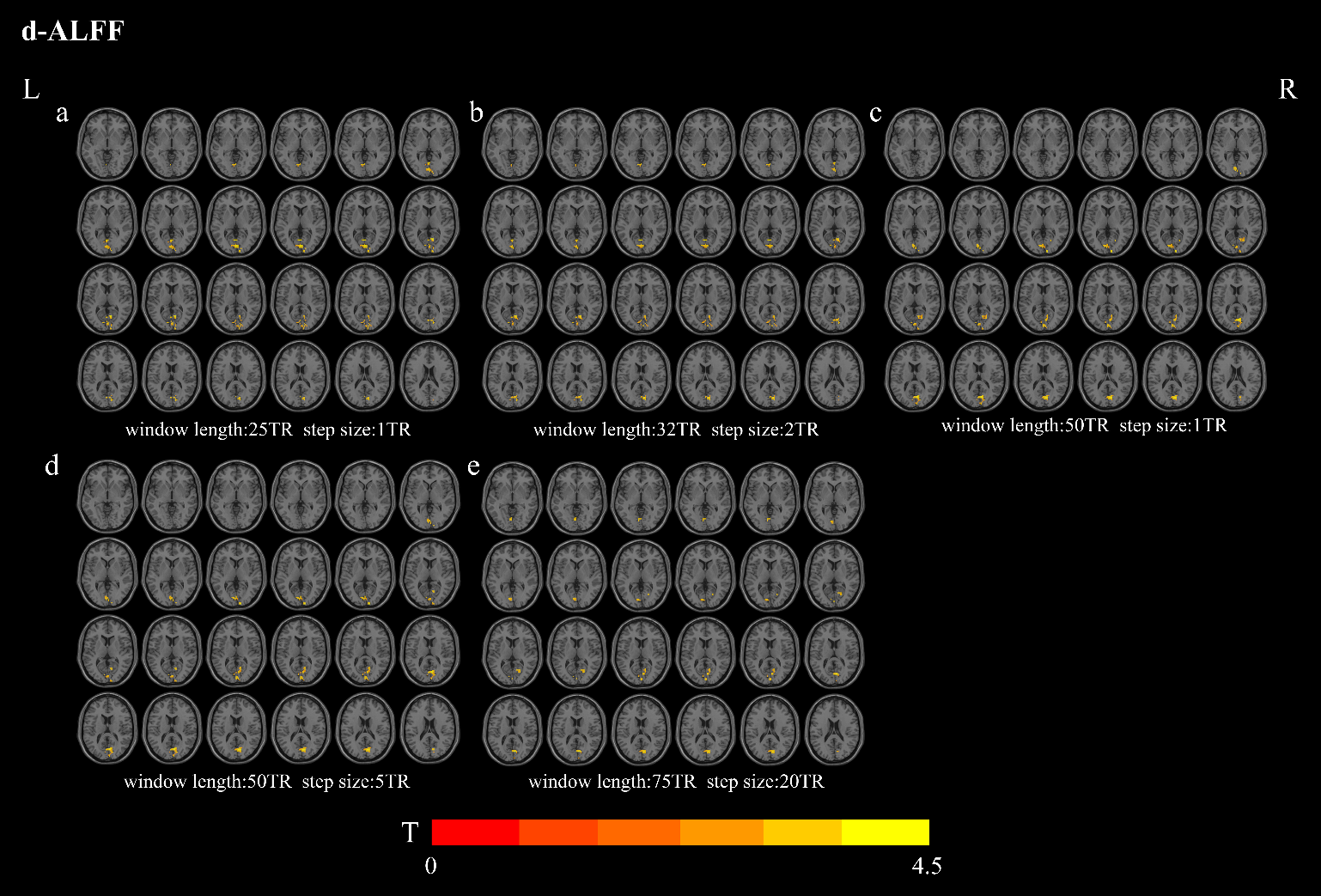


FIGURE S4 | Brain regions with significant differences in d-ALFF between TIA group and HCs group (after GRF correction; voxel-wise p < 0.005, cluster-wise p < 0.05, two-tailed) under different window lengths. Parameter: smooth kernel = 6 mm. The color bar indicates the T-value. d-ALFF, dynamic amplitude of low-frequency fluctuations; L, left; R, right.


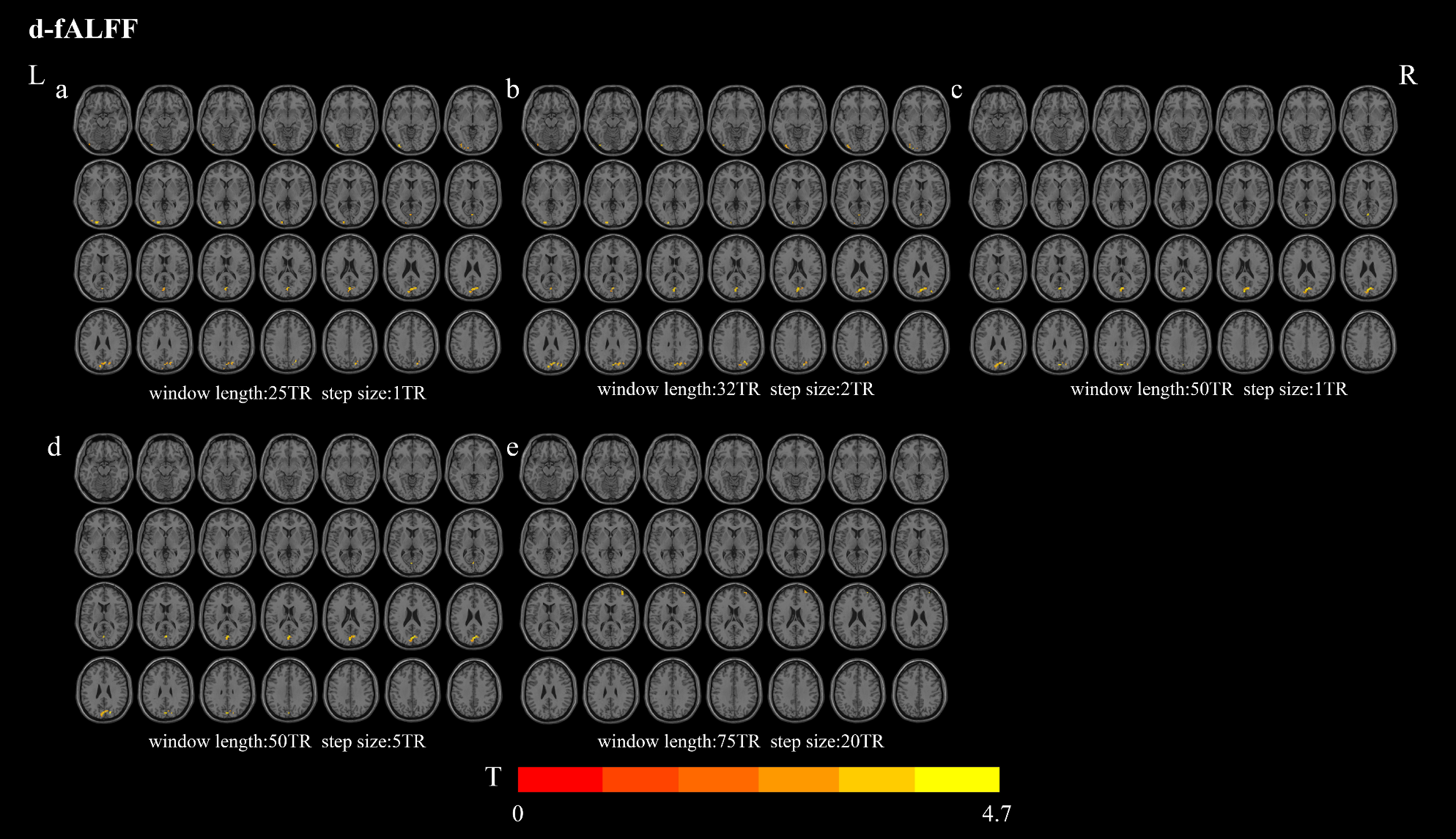


FIGURE S5 | Brain regions with significant differences in d-fALFF between TIA group and HCs group (after GRF correction; voxel-wise p < 0.005, cluster-wise p < 0.05, two-tailed) under different window lengths. Parameter: smooth kernel = 6 mm. The color bar indicates the T-value. d-fALFF, dynamic fractional amplitude of low-frequency fluctuations; L, left; R, right.


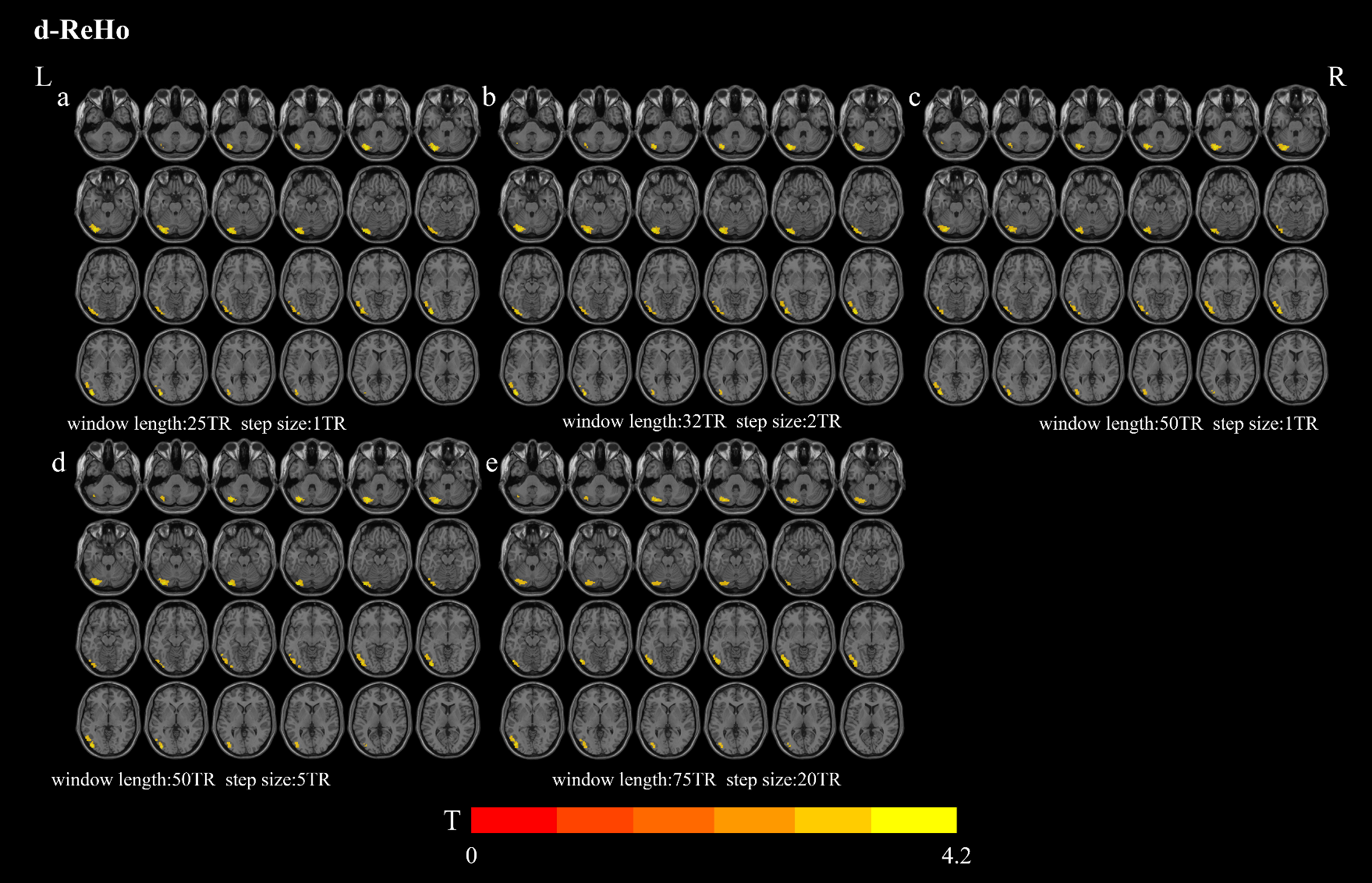


FIGURE S6 | Brain regions with significant differences in d-ReHo between TIA group and HCs group (after GRF correction; voxel-wise p < 0.005, cluster-wise p < 0.05, two-tailed) under different window lengths. Parameter: smooth kernel = 6 mm. The color bar indicates the T-value. d-ReHo, dynamic regional homogeneity; L, left; R, right.


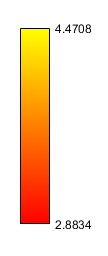

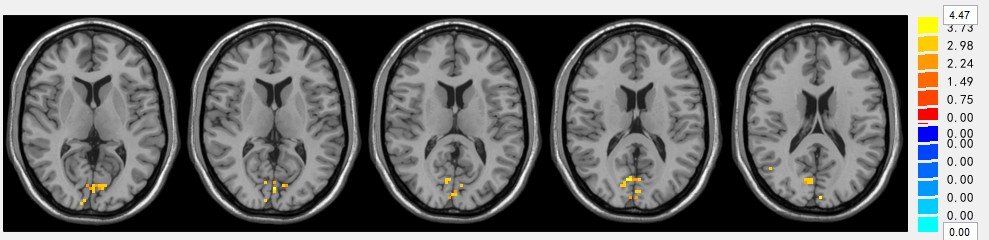


FIGURE S7 | Group differences of the d-ALFF after mean FD regressed out.


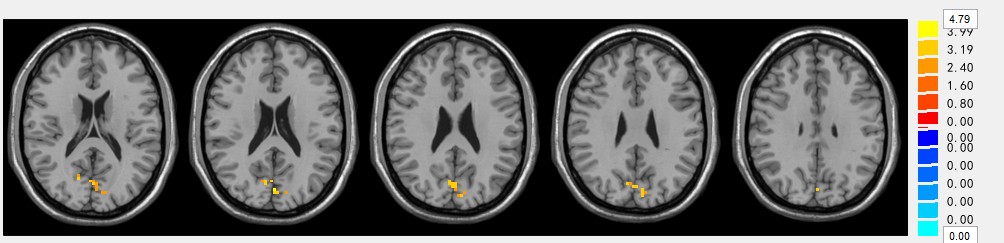

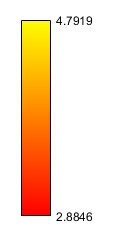


FIGURE S8 | Group differences of the d-fALFF after mean FD regressed out.


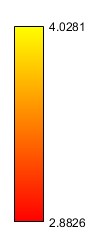

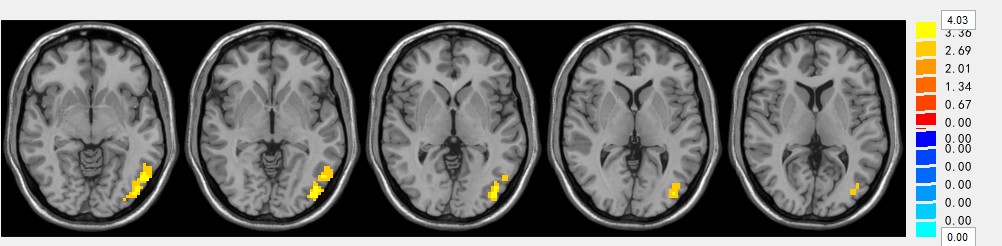


FIGURE S9 | Group differences of the d-ReHo after mean FD regressed out.


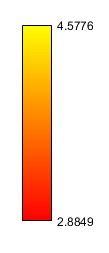

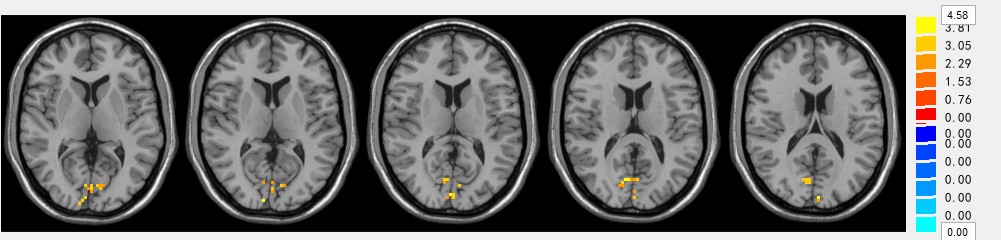


FIGURE S10 | Group differences of the d-ALFF after age and gender regressed out.


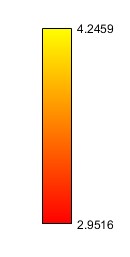

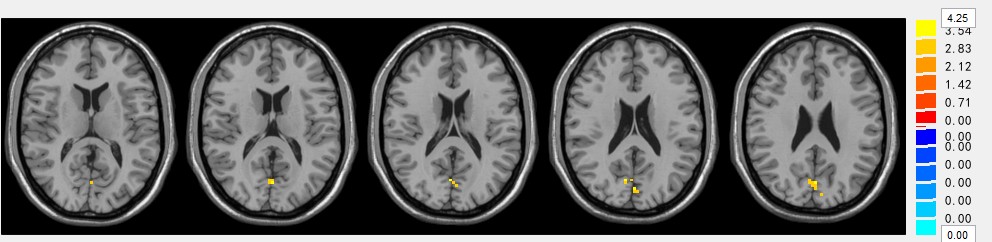


FIGURE S11 | Group differences of the d-fALFF after age and gender regressed out.


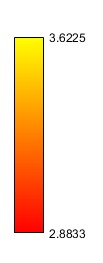

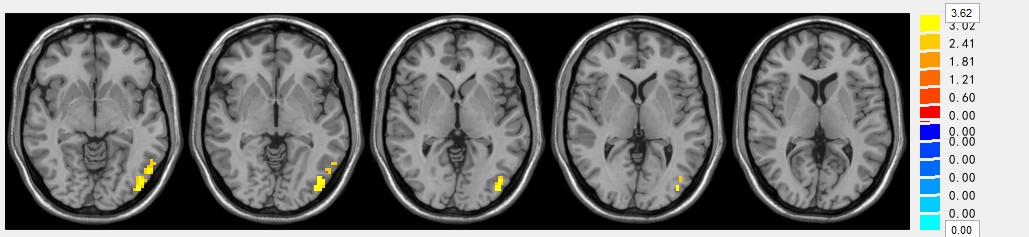


FIGURE S12 | Group differences of the d-ReHo after age and gender regressed out.


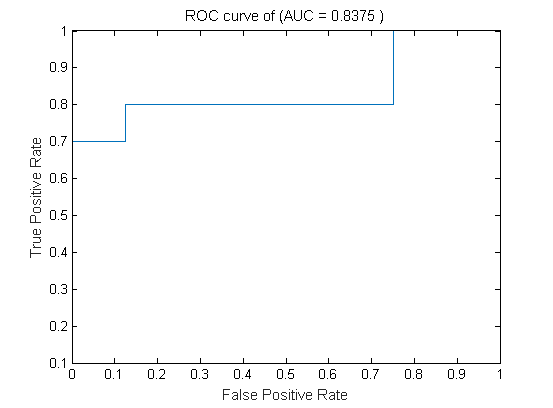


FIGURE S13 | The receiver operating characteristic (ROC) curve of dynamic metrics. Using subset of optimal features in SVM, we classified TIA from HCs with an accuracy, sensitivity, specificity, precision, and the area under the receiver operating characteristic (ROC) curve of 83.3%, 70%, 1, 1 and 0.8375, respectively.
